# Reevaluating Axillary Lymph Node Dissection in Total Mastectomy for Low Axillary Burden Breast Cancer: Insights from a Meta-Analysis including the SINODAR-ONE Trial

**DOI:** 10.3390/cancers16040742

**Published:** 2024-02-10

**Authors:** Munaser Alamoodi, Neill Patani, Kinan Mokbel, Umar Wazir, Kefah Mokbel

**Affiliations:** 1 The London Breast Institute, Princess Grace Hospital, 42-52 Nottingham Place, London W1U 5NY, UK; malamoodi@kau.edu.sa (M.A.); neill.patani@hcahealthcare.co.uk (N.P.); k.mokbel@ex.ac.uk (K.M.); umar.wazir@rcsed.ac.uk (U.W.); 2 Department of Surgery, King Abdulaziz University, Jeddah 21589, Saudi Arabia; 3 Institute for Women’s Health, University College London, London WC1N 1DZ, UK; 4 Medical School, University of Exeter, Exeter EX1 2LU, UK

**Keywords:** mastectomy, node-positive breast cancer, axillary lymph node dissection, axillary dissection, sentinel lymph node biopsy, targeted axillary dissection, post-mastectomy radiotherapy, axillary radiotherapy, overall survival, recurrence-free survival

## Abstract

**Simple Summary:**

In the past, breast cancer patients with lymph node involvement often had surgery to remove most or all of the lymph nodes in the armpit, which can cause significant side effects. However, recent research, like the ACOSOG Z0011 study, has shown that for patients with only a few affected lymph nodes who are getting breast-conserving treatment and radiation treatment, this extensive surgery might not be necessary and doesn’t affect their chances of survival. This study looked at whether the same applies to patients having a total mastectomy. We analyzed several studies involving over 4000 patients and found that skipping the extensive lymph node surgery didn’t impact overall survival after about 7 years of follow-up. This suggests that certain breast cancer patients having mastectomy with limited lymph node involvement, can safely avoid the more invasive surgery for the lymph nodes in armpit. However, more research is needed to fully understand how other factors might affect survival.

**Abstract:**

Complete axillary lymph node dissection (cALND) was previously the standard of care for breast cancer (BC) patients with axillary node disease or macro-metastases found via sentinel lymph node biopsy (SLNB). However, due to significant morbidity, contemporary management now considers a more selective approach, influenced by studies like ACOSOG Z0011. This trial showed that cALND could be omitted without compromising local control or survival in patients with low axillary nodal disease burden undergoing breast-conserving therapy, radiotherapy, and systemic therapy. The relevance of this approach for women with low axillary nodal burden undergoing total mastectomy (TM) remained unclear. A PubMed search up to September 2023 identified 147 relevant studies, with 6 meeting the inclusion criteria, involving 4184 patients with BC and low-volume axillary disease (1–3 positive lymph nodes) undergoing TM. Postmastectomy radiotherapy receipt was similar in both groups. After a mean 7.2-year follow-up, both the pooled results and the meta-analysis revealed no significant differences in overall survival. The combined analysis of the published studies, including the subgroup analysis of the SINODAR-One trial, indicates no survival advantage for cALND over SLNB in T1–T2 breast cancer patients with 1–3 positive sentinel lymph nodes (pN1) undergoing mastectomy. This suggests that, following a multidisciplinary evaluation, cALND can be safely omitted. However, the impact of other patient, tumor, and treatment factors on survival requires consideration and therefore further prospective trials are needed for conclusive validation.

## 1. Introduction

For over a century, breast cancer (BC) surgery adhered to the Halstedian model, viewing it as a primarily locoregional condition spreading via the lymphatic system, curable through extensive surgical resection. Nonetheless, it is now evident that BC’s biological attributes, rather than surgical extent, predominantly influence the risk of both systemic and locoregional recurrence (LRR) [1]. This shift has prompted the adoption of less invasive strategies, enhancing clinical outcomes and patient quality of life [2].

Complete axillary lymph node dissection (cALND), historically the gold standard for identifying axillary nodal metastases in clinically node-negative early BC patients, has been replaced by sentinel lymph node biopsy (SLNB) as a staging procedure. Tracer-guided lymphatic mapping techniques and SLNB standardization have substantially reduced cALND frequency, establishing SLNB as the standard for clinically node-negative patients at diagnosis [3,4]. For many years, cALND was routinely recommended for patients with biopsy-proven nodal disease or macro-metastases identified by SLNB to optimize locoregional control and comprehensive axillary staging. However, cALND carries a notable risk of morbidity, including limited shoulder mobility, potential nerve injury, chronic pain, and lymphedema, all detrimentally impacting quality of life [3,4].

The ACOSOG Z0011 trial reshaped the management of patients with positive sentinel nodes, demonstrating that those with limited sentinel lymph node metastases did not benefit from subsequent cALND. Importantly, SLNB alone, compared to subsequent cALND, showed no inferior survival or significantly higher local recurrence rates, albeit for women undergoing BCS, adjuvant RT, and systemic therapy [5]. This landmark study set the stage for safely omitting cALND in select patients undergoing BCS and established a precedent for axillary surgery de-escalation. A recent meta-analysis has corroborated these findings in the context of BCS [6]. However, these data could not directly apply to patients with limited lymph node disease undergoing mastectomy rather than BCS. This knowledge gap created a dilemma for the mastectomy cohort, leaving many questions unanswered and sparking ongoing controversy. The 2017 American Society of Clinical Oncology (ASCO) guidelines acknowledged this issue and concluded that additional axillary surgery beyond SLNB should be offered to women with early BC and 1–2 sentinel lymph node metastases treated with mastectomy. The primary reason was insufficient evidence [7]. However, the 2021 ASCO guidelines suggested that further axillary surgery can be omitted in this setting but described the level of evidence as weak. [8]. Nevertheless, it is expected that the de-escalation of cALND will likely expand to include mastectomy patients, with practice guidelines evolving as new evidence emerges [9]. While numerous retrospective studies have addressed this important issue, the SINODAR-ONE trial subgroup analysis study remains the sole prospective trial to date [10]. The interim subgroup analysis of this trial has recently reported similar survival outcomes in patients who underwent TM with 1–2 positive lymph nodes, comparing cALND to SLNB-only patients. It offers an enticing prospect of safely omitting cALND in mastectomy patients with low axillary disease burden.

Our review and meta-analysis aim to validate interim prospective findings by incorporating them into the broader context of retrospectively published studies. However, limitations arise from the retrospective nature of many studies in our analysis and the absence of detailed information on adjuvant systemic and radiation therapy.

## 2. Materials and Methods

### 2.1. Literature Search

A PubMed database search was conducted by two authors up to September 2023. The search criteria involved terms such as mastectomy with axillary lymph node dissection, mastectomy with sentinel lymph node biopsy, and mastectomy with 1–3 involved lymph nodes. The study cohort was required to encompass both ALND and SLNB subgroups, accompanied by relevant data on OS and/or recurrence-free survival (RFS). Studies were considered for inclusion if they presented data for both TM and BCS, provided that sufficient data for the mastectomy subgroup were available. Exclusion criteria encompassed studies related to node-negative disease and those exclusively focused on the role of axillary surgery in patients undergoing BCS. Additionally, case reports, correspondence, editorials, perspectives, and studies not published in English were excluded. In our analysis, the quality of the five nonrandomized studies was assessed using the Newcastle–Ottawa Scale (NOS), which provides a quality score ranging from 0 to 9 [11].

### 2.2. Data Extraction and Analysis

The data were subsequently collected and amalgamated into two distinct groups, each exclusively composed of patients treated with TM and having 1–3 positive axillary lymph nodes. When hazard ratios (HR) were not explicitly provided, they were estimated based on the data reported in the original publications. To conduct the meta-analysis and generate forest plots, the statistical analysis program ‘STATA’ (STATA Corporation, College Station, TX, USA) was used. The assessment of patients who underwent RT was conducted across each study, and the percentage of the total patient population was computed for both the cALND and SLNB groups. For appropriate cases, the chi-square test with Yates’s correction was applied. To gauge heterogeneity, measures were calculated from the data, and confidence intervals (CI) were determined based on a non-central chi-square distribution for Q (a common effect measure).

## 3. Results

**Figure 1:**
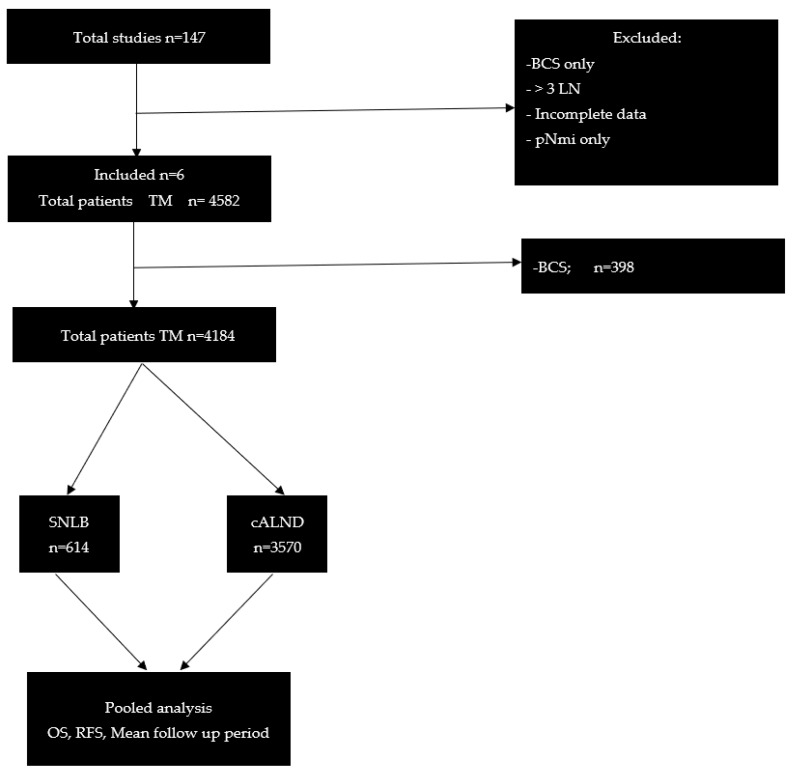
Selection of studies and distribution of patients (CONSORT flow diagram). Abbreviations: Total mastectomy (TM), breast conserving surgery (BCS), sentinel lymph node biopsy (SLNB), complete axillary lymph node dissection (cALND), lymph node (LN), Metastasis greater than 0.2 mm and/or more than 200 cells, but none greater than 2.0 mm (pNmi).

**Figure 2:**
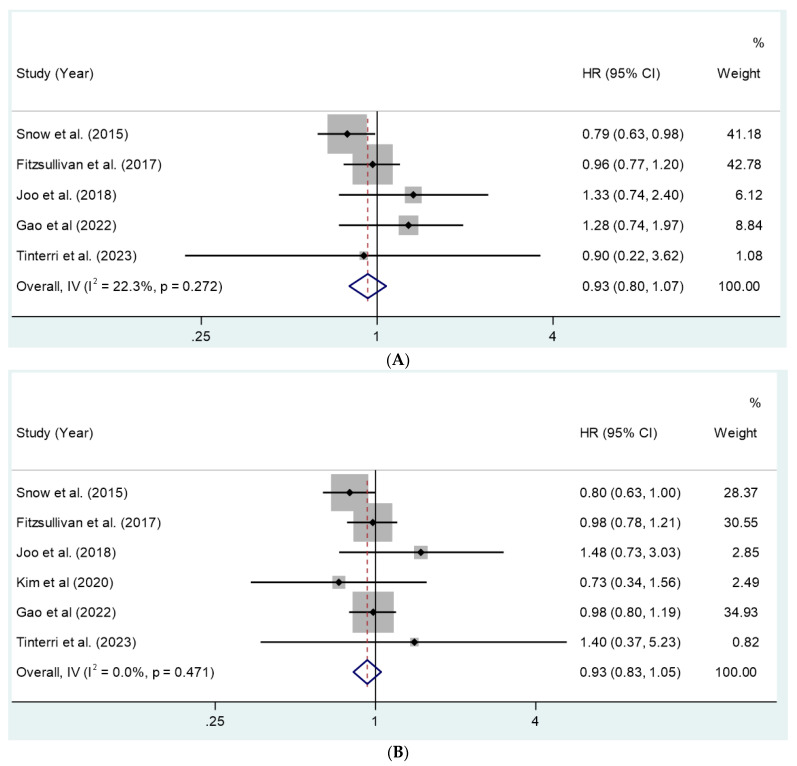
(**A**) Forest plot of RFS for cALND vs. SLNB. (**B**) Forest plot of OS for cALND vs. SLNB. Note: Individual and pooled hazard ratios (HRs) from studies were reported in the forest plot. Squares represent study-specific estimates, and the size of the square reflects the study-specific weight (i.e., the inverse of the variance). The diamond represents the summary estimate with a 95% confidence interval (CI), and the horizontal lines indicate 95% confidence intervals. An HR below 1 favors SLNB and that above 1 favors cALND [10,12,13,14,15,16].

The search resulted in 147 papers, of which 6 met the inclusion criteria [10,12,13,14,15,16] (Table 1). Figure 1 provides a detailed illustration of the study selection and patient distribution. Evaluation using the NOS for the five nonrandomized studies included in our analysis indicated a score of four in two studies [12,13] and five out of nine in three studies [14,15,16]. Hence, all the nonrandomized studies assessed were considered to be of fair quality according to NOS standards [11]. The total number of patients included in the analysis was 4184. They were categorized into groups as follows: TM with cALND (*n* = 3570), TM with SLNB (*n* = 614), TM with cALND plus post-mastectomy radiotherapy (PMRT) (*n* = 628), and TM with SLNB plus PMRT (*n* = 73). Patients in the cALND group had a mean age of 50 years [95% CI: 49.6–50.1], while those in the SLNB group had a mean age of 52 [95% CI: 51–52] (*p* = 0.607). Most patients analyzed had T1–T2 tumors, with only a small proportion (9.6%) having T3–T4 tumors. The majority had macro-metastasis in the initial SLNB, and a smaller percentage (7.2%) had micro-metastasis. Data on PMRT were available from four studies [10,12,15,16]. The frequency of receiving PMRT did not significantly differ between the cALND and SLNB groups (26.01% vs. 20.0%, *p* = 0.0593) (Table 2). One study reported OS but not RFS [13]. The pooled mean follow-up period was 7.2 years. Pooled OS rates were calculated to be 88.9% [95% CI: 88.5–89.3] for cALND and 93.2% [95% CI: 92.7–93.7] for SLNB. The pooled RFS rates were 86.01% [95% CI: 85.5–86.5] for cALND and 89.7% [95% CI: 88.7–90.7] for SLNB. No statistically significant differences in age, OS, or RFS were observed between patients who underwent cALND and those who underwent SLNB (*p* = 0.472 and 0.605, respectively). The meta-analysis, pooling aggregate data using the common-effect inverse variance model, revealed a combined HR of 0.931 (95% CI: 0.826–1.050) for OS (six studies) and 0.929 (95% CI: 0.803–1.074) for RFS (five studies). Figure 2A,B represent the corresponding forest plots. The meta-analysis showed no significant heterogeneity among the analyzed studies.

## 4. Discussion

This is the first comprehensive analysis of axillary surgery in patients treated with mastectomy for BC with 1–3 positive lymph nodes that incorporates data from the subgroup analysis of the recently reported randomized controlled trial (SINODAR-ONE). The meta-analysis of survival outcomes includes a substantial number of patients (*n* = 4184) with a meaningful duration of clinical follow-up (mean 7.2 years). In this selected cohort, the burden of axillary disease in the initial SLNB was relatively low, with up to three involved nodes. Although a direct comparison of primary tumor characteristics and systemic therapy between the two groups was hindered by data limitations, the standardization of surgical treatment through mastectomy and a similar extent of node involvement likely resulted in no significant differences in these parameters. This is especially true since systemic therapy recommendations based on estrogen receptor and HER2 expression are likely to be similar regardless of the total lymph node burden, as dictated by international guidelines during the study periods. Patients in the cALND group were, on average, 1.8 years younger than those in the SLNB group, suggesting a potential underlying bias toward recommending cALND in younger patients. Furthermore, the majority of patients had T1–T2 tumors, and the receipt of PMRT was similar between the two groups. The results demonstrate that those who underwent SNLB alone did not have inferior OS or RFS outcomes compared to those who underwent cALND. This aligns with established evidence from randomized controlled trial ACOSOG Z0011 indicating that cALND does not enhance OS in patients undergoing BCS with limited involvement of the sentinel nodes [5].

These observations are underpinned by a growing body of evidence suggesting that invasive BC, even in its earliest stages, is inherently a systemic disease. This concept finds support in the presence of circulating tumor cells (CTCs) in the majority of patients with invasive BC [17], signifying that early invasive BC often exhibits systemic characteristics from the outset. Moreover, research indicates that the tumor microenvironment (TME) extends beyond the primary tumor site to encompass the chest wall and regional lymph nodes. Recent studies propose that lymph node microenvironment can provide an accommodating niche for cancer cell colonization and growth. Within the lymph nodes, immune and stromal cells express and release molecules that may facilitate cancer cell survival [18]. The upregulation of the CCR7/CCL21 axis appears to guide reactivated CCR7 + CTCs toward regional lymph nodes [19]. Consequently, in contrast to systemic therapy, the surgical removal of residual microscopic disease in the axillary lymph nodes is unlikely to confer a survival benefit.

The absence of a survival advantage provided by cALND should be integrated into the informed decision-making process for breast cancer patients. This is especially crucial given the substantial morbidity associated with cALND, such as pain, numbness, restricted shoulder mobility, and lymphedema, which can significantly impact patients’ quality of life [20]. Nevertheless, it is noteworthy that the AMAROS trial did indicate a potential reduction in regional recurrence with cALND compared to SLNB followed by RT, however there was no significant difference in OS [20]. The compiled survival outcomes in our study align with the SINODAR-ONE trial data and encompass findings from the previously published literature. The SINODAR-ONE trial is a prospective, multi-center randomized non-inferiority study that explores the role of cALND in patients undergoing T1–T2 BC treatment with one or two macro-metastatic sentinel lymph nodes, whether through BCS or TM [21]. In a recent subgroup analysis conducted by Tinterri et al. [10] using the SINODAR-ONE trial data, no statistically significant differences were observed in OS (*p* = 0.597) or RFS (*p* = 0.821) for TM patients who underwent either cALND or SLNB. It is important to note that this trial is currently the sole prospective randomized study that has reported on this specific patient group. However, it is essential to acknowledge that the SINODAR-ONE study focusing on mastectomy patients has inherent limitations. The potential for false-negative outcomes is evident in the subgroup analysis due to the considerably smaller sizes of the two treatment cohorts compared to the original study population. The statistical robustness of the analysis may have been further compromised by suboptimal accrual rates and a scarcity of anticipated events. Furthermore, although disparities in PMRT rates were observed between the treatment arms, comprehensive adjuvant radiotherapy (RT) data are currently unavailable. Lastly, the study exhibits a relatively brief follow-up duration. To reinforce the conclusions of the trial, the recruitment of BC patients with one to two positive sentinel lymph nodes selected for mastectomy was reinitiated. Several retrospective studies have also corroborated these findings [12,13,14,15,16]. FitzSullivan et al. [14] analyzed 525 patients who underwent cALND and SLNB and found no reported difference in OS among the four groups studied, which included SLNB only, SLNB with RT, cALND only, and cALND with RT. The incidence of LRR was not significantly different for patients who received no further axillary treatment compared to those who underwent cALND without RT or those treated with RT without cALND (10-year recurrence rate: 3.8% vs. 1.6% and 0%, respectively). RFS and OS were not significantly different among patients who received no further axillary treatment compared to those who underwent cALND, RT, or both. In a meta-analysis of ten studies conducted by Gao et al. [15] involving women with 1–2 positive lymph nodes undergoing either BCS or TM, cALND did not influence disease outcomes for those with 1–2 positive nodes treated with TM. The survival benefit of cALND remained non-significant after restricting the analysis to four studies with patients who had BCS or three studies with patients who had TM. In a study that included 1697 patients undergoing TM, Joo et al. [16] similarly demonstrated no significant differences in RFS and OS. Importantly, the authors identified other factors influencing survival, including tumor location, size, number of metastatic lymph nodes, hormone receptor status, histologic grade, body mass index, and chemotherapy. The only adjuvant treatment significantly associated with OS and RFS was hormone therapy, likely due to the worse prognosis of hormone-insensitive tumors. Arisio et al. [22] reported, in a multivariate analysis, that biological features were significant independent predictors of poor OS and RFS, suggesting that tumor biology, rather than the extent of axillary or breast surgery, has a predominant impact on prognosis. When compared with contemporary standards of treatment, it is important to highlight that a significant number of patients in the analyzed studies did not receive optimal systemic therapy. For instance, the Sinodar study included patients with Her2-positive or triple-negative breast cancer who did not receive the gold standard NST [10].

In addition to tumor biology, systemic therapy, and patient characteristics, the clinical outcome in BC can be potentially influenced by RT. The AMAROS trial conducted in 2014 directly compared cALND to dedicated axillary treatment with RT in node-positive breast cancer patients. The study showed that RT led to significantly lesser morbidity rates and achieved similar OS and comparable axillary control for patients with primary T1–T2 breast cancer and no palpable lymphadenopathy, including those undergoing TM [20]. In a similar vein, Fu et al. [23] concluded that radiation was as effective as cALND in patients with mastectomy and pN1 disease in terms of OS and RFS, with radiation after SLNB having fewer side effects compared to cALND. Moreover, a recent meta-analysis encompassing findings from five randomized controlled trials indicated that after cALND, regional node irradiation (RNI) led to marginal improvements in breast cancer-specific mortality, RNI, and distant metastases-free survival, but it did not confer an advantage in OS. In the context of a positive SLNB, RNI may provide equivalent locoregional control and disease-free survival (DFS) compared to cALND, with the added benefit of a reduced risk of lymphedema. It is important to note that no randomized data are currently available for LRNI in the neoadjuvant setting [24]. As a result, SLNB followed by RT could be considered an alternative to cALND in patients with mastectomy and pN1 disease identified by SLNB.

PMRT was found not to improve OS in patients with isolated tumor cells or micromeastasis in the axillary lymph nodes [25]. While PMRT is the current standard of care for patients with 4 or more involved axillary lymph nodes, the indications for PMRT in patients with 1–3 involved nodes remain a subject of debate [26]. Previously, we reported that in breast cancer patients with 1–3 positive lymph nodes, PMRT significantly reduced the risk of LRR and was associated with a modest OS benefit [27]. Pending the publication of results from ongoing randomized controlled trials, PMRT should be considered in this patient group following a comprehensive multidisciplinary discussion. Most patients in this pooled analysis had stage T1–T2 breast cancer, which may explain the favorable survival outcomes without adjuvant RT. Interestingly, systemic therapy may also influence the impact of adjuvant radiation. Patients with T3 disease receiving chemotherapy, either in the neoadjuvant or adjuvant setting, do not appear to benefit from PMRT. However, OS can be improved by PMRT in patients not receiving chemotherapy [28]. The question of PMRT’s role in patients with 1–3 positive nodes is currently under investigation in an ongoing randomized controlled trial [29]. Therefore, it is highly unlikely that radiation played a role in the non-inferiority of SLNB compared to cALND concerning OS.

Contemporary clinical practice has seen an increasing number of patients with positive axillary nodes diagnosed before surgery and recommended for tailored NST. Many of these patients achieve a pathological complete response (pCR). This trend has led to a reduction in the extent of axillary surgery. A recent meta-analysis demonstrated that there is no significant benefit of cALND over TAD in patients with axillary pCR [30]. According to the recently presented data from the NRG Oncology/NSABP B-51/RTOG 1304 study, omitting RNI appears safe in many patients who convert from cN1 to ypN0 after NST.

The strength of our analysis lies in the extensive patient cohort included in the study. Nevertheless, there are several limitations to consider. Firstly, the majority of the studies included in our analysis were retrospective in nature, which introduces potential heterogeneity regarding varying systemic and radiation treatments. Additionally, it is crucial to acknowledge the potential impact of selection bias when interpreting the conclusions of our pooled analysis, as patients undergoing cALND often exhibit poorer prognostic features compared to those undergoing SLNB only. The smaller patient population in the SLNB group compared to the cALND group presents another limitation, potentially indicating a selection bias and adherence to prevailing standard practice guidelines [7]. Furthermore, it is important to note that although most patients included in the analysis had macro-metastases in their sentinel lymph nodes, some had micro-metastatic disease, which generally carries a more favorable prognosis than macro-metastases [31].

In addition to the ongoing extended investigation of the Sinodar-One trial, there are other prospective trials specifically addressing the safety of omitting cALND in patients with 1–2 macro-metastatic nodes. The ongoing POSNOC trial aims to recruit 1900 patients, eligible women with T1/T2 invasive breast cancer with 1 or 2 macro-metastases at sentinel node biopsy. The intervention group receives adjuvant therapy alone, while the standard care group undergoes cALND or axillary RT. Both groups receive adjuvant therapy according to local guidelines, including systemic therapy and, if necessary, RT to the breast or chest wall [32]. These studies should provide robust data supporting the safety of SLNB alone in women with one or two SLN macro-metastases treated with BCS or TM.

## 5. Conclusions

Our study demonstrates that there is no survival advantage for cALND over SLNB in patients with T1–T2 breast cancer and 1–3 positive sentinel lymph nodes (pN1) undergoing mastectomy. This suggests that, following thorough multidisciplinary evaluation, cALND can be safely omitted in these patients. Nevertheless, it is essential to consider the potential impact of other patient-specific, tumor-related, and treatment-related factors on survival outcomes. Therefore, adequately powered prospective trials are warranted to validate these findings conclusively.

## Figures and Tables

**Table 1:** Patient distribution and pooled analysis. * The patients from Kim et al. were removed from the total patient for cALND and SLNB to calculate the RFS. Abbreviations: complete axillary lymph node dissection (cALND), sentinel lymph node biopsy (SLNB).

Reference	Author (Year)	Total Patients = *n*	cALND Group = *n*	SLNB Group = *n*	Overall Survival cALND (%)	Overall Survival SLNB (%)	Recurrence-Free Survival cALND (%)	Recurrence-Free Survival SLNB (%)
[12] Retrospective	Snow et al. (2015)	189	157	32	90.0 (*n* = 141) (5 years)	100 (*n* = 32) (5 years)	85 (*n* = 133) (5 years)	100 (*n* = 32) (5 years)
[14] Retrospective	Fitzsullivan et al. (2017)	525	455	70	86.0 (*n* = 391) (10 years)	87.9 (*n* = 62) (10 years)	82.2 (*n* = 374) (10 years)	84.9 (*n* = 59) (10 years)
[16] Retrospective	Joo et al. (2018)	1697	1539	158	84.0 (*n* = 1293) (10 years)	84.0 (*n* = 133) (10 years)	83.0 (*n* = 1277) (10 years)	83.0 (*n* = 131) (10 years)
[13] Retrospective	Kim et al. (2020)	883	704	179	91.8 (646) (4.5 years)	95.5 (*n* = 171) (4.5)	-	-
[15] Retrospective	Gao et al. (2022)	672	604	68	98.0 (*n* = 592) (3 years)	100 (*n* = 68) (3 years)	95.2 (*n* = 575) (3 years)	98.5 (*n* = 67) (3 years)
[10] Prospective	Tinterri et al. (2023)	218	111	107	97.8 (*n* = 109) (5 years)	98.7 (*n* = 106) (5 years)	95.7 (*n* = 106) (5 years)	94.1 (*n* = 101) (5 years)
Total		4184	3570	614	3172 (88.9%)	572 (93.2%)	2465 (86.01%) *	390 (89.7%) *

**Table 2:** PMRT (post-mastectomy radiotherapy) patient distribution. cALND = complete axillary lymph node dissection. SLNB = sentinel lymph node biopsy, - incomplete data.

Reference	Author (Year)	cALND Patients = *n*	SNLB Patients = *n*	cALND + PMRT	SLNB + PMRT
[12] Retrospective	Snow et al. (2015)	157	32	71	13
[14] Retrospective	Fitzsullivan et al. (2017)	-	-	-	-
[16] Retrospective	Joo et al. (2018)	1539	158	148	14
[13] Retrospective	Kim et al. (2020)	0	0	0	0
[15] Retrospective	Gao et al. (2022)	604	68	379	38
[10] Prospective	Tinterri et al. (2023)	111	107	30	8
Total (%)		2411	365	628 (26.01)	73 (20.0)

## Data Availability

The datasets generated in this study are publicly available in this open access publication without any restrictions.
